# Supplementary material for: Quantitative measurement of diffusion-weighted imaging signal using expression-controlled aquaporin-4 cells: Comparative study of 2-compartment and diffusion kurtosis imaging models
Source: PLoS One. 2022 Apr 19;17(4):e0266465. doi: 10.1371/journal.pone.0266465 (PMC9017930; doi:10.1371/journal.pone.0266465)
Supplement: Attachment — Submitted filename: Response to Reviewers.docx [file pone.0266465.s002.docx]

Thank you very much for your helpful comments concerning our manuscript: PONE-D-21-38062.

According to the comments, the manuscript was revised as follows.

Journal Requirements

1. Please ensure that your manuscript meets PLOS ONE's style requirements, including those for file naming. The PLOS ONE style templates can be found at

https://journals.plos.org/plosone/s/file?id=wjVg/PLOSOne_formatting_sample_main_body.pdf and

https://journals.plos.org/plosone/s/file?id=ba62/PLOSOne_formatting_sample_title_authors_affiliations.pdf

(JR-1-0)

Referring to the attached file, the manuscript body was fixed to "justified". (Page 2, Line 16, etc.)

(JR-1-1)

Referring to the PLOS ONE TITLE, AUTHOR, AFFILIATIONS FORMATTING GUIDELINES, title was fixed to “centered”. (Page 1, Line 1-3.)

(JR-1-2)

Referring to the PLOS ONE MANUSCRIPT BODY FORMATTING GUIDELINES, paragraphs were “indented”. (Page 2, Line 17, etc.)

(JR-1-3)

In Figs 2-4, figure titles were corrected to 15 characters or less, and figure legends were changed as well. (Fig 2; Page 9, Line 141-146. Fig 3; Page 9, Line 147 - Page 10, Line 153. Fig 4; Page 10, Line 158 – 166.)

(JR-1-4)

All equations were “centered”. (Page 7, Line 102 & 109.)

(JR-1-5)

The text in Table 1 was fixed to “justified” and “double-spaced”. (Page 11, Line 169.)

(JR-1-6)

The style of the “References” was corrected. (Page 16, Line 242 – Page 18, Line 297.)

2. PLOS requires an ORCID iD for the corresponding author in Editorial Manager on papers submitted after December 6th, 2016. Please ensure that you have an ORCID iD and that it is validated in Editorial Manager. To do this, go to ‘Update my Information’ (in the upper left-hand corner of the main menu), and click on the Fetch/Validate link next to the ORCID field. This will take you to the ORCID site and allow you to create a new iD or authenticate a pre-existing iD in Editorial Manager. Please see the following video for instructions on linking an ORCID iD to your Editorial Manager account: https://www.youtube.com/watch?v=_xcclfuvtxQ

(JR-2)

The corresponding author has the following ORCID iD: 0000-0002-2949-4687.

3. Thank you for stating in your Funding Statement:

(This work was supported by grants from KAKENHI (15H04910, TO received.).

The funders had no role in study design, data collection and analysis, decision to publish, or preparation of the manuscript.)

Please provide an amended statement that declares *all* the funding or sources of support (whether external or internal to your organization) received during this study, as detailed online in our guide for authors at http://journals.plos.org/plosone/s/submit-now. Please also include the statement “There was no additional external funding received for this study.” in your updated Funding Statement.

Please include your amended Funding Statement within your cover letter. We will change the online submission form on your behalf.

(JR-3)

Our Funding Statement was amended according to your comment. One other grant was added (KAKENHI (20K08150, JK received.) and the Ministry of Education, Culture, Sports, Science and Technology (MEXT), Japanese government) to the cover letter.

4. Please review your reference list to ensure that it is complete and correct. If you have cited papers that have been retracted, please include the rationale for doing so in the manuscript text, or remove these references and replace them with relevant current references. Any changes to the reference list should be mentioned in the rebuttal letter that accompanies your revised manuscript. If you need to cite a retracted article, indicate the article’s retracted status in the References list and also include a citation and full reference for the retraction notice.

(JR-4)

The style of the reference list was corrected. (Page 16, Line 242 – Page 18, Line 297.) There are no retracted references in the list. According to Reviewer #1’s comment, the introduction was revised and references were added [4, 6, 7, 9-11].

Reviewers' comments

5. Review Comments to the Author

Reviewer #1: This manuscript is aimed at a very important aspect of DWI - understanding the relationship between biological tissue structure and the detected signal. The authors compared 2Comp and DKm models obtained from DWI of AQP4 expressing and non-expressing cells to look for biomarkers that indicate differences in cell membrane water permeability. The idea is straightforward and while the authors acknowledge two significant limitations (one being the fact that theAQP4 expression was not measured, which would have allowed for a more polished analysis with precise correlation between the expression level of AQP4 and the DWI parameters, the second being the in vitro aspect), the paper is nonetheless important to promote discussion and advance the field. To really emphasize the clinical implications of identifying biomarkers in this space, augmenting the intro to reflect the eventual clinical importance of this study, may be of value outside of the basic science context/audience.

(CA-5-R1)

In response to the reviewer’s comment, the introduction has been revised to emphasize the clinical importance of this study. (Page3, Line 42 – 53.)

“Aquaporin channels are known to be associated with various diseases. For example, neuromyelitis optica (NMO) is an autoimmune disease targeting AQP4 [3]. AQP4 is also associated with brain tumors [4] and neurodegenerative diseases including Alzheimer's disease [5]. It is also known that changes to the expression of AQP4 can alter the accumulation of brain edema in ischemia [6, 7]. Other aquaporin subtypes are involved in various diseases such as tumors [8], cataracts [9], and nephrogenic diabetes insipidus [10, 11]. Unfortunately, a clinical imaging method that can evaluate aquaporin expression *in vivo* has not yet been established. Moreover, as there are many subtypes of aquaporin with diverse functions, it is probably more reasonable to evaluate the cell membrane water permeability rather than the expression of aquaporin itself. It is therefore expected that an imaging technique that can quantitatively evaluate changes in cell membrane water permeability will be useful in disease diagnosis.”
